# Pharmacological Inhibition of FTO

**DOI:** 10.1371/journal.pone.0121829

**Published:** 2015-04-01

**Authors:** Fiona McMurray, Marina Demetriades, WeiShen Aik, Myrte Merkestein, Holger Kramer, Daniel S. Andrew, Cheryl L. Scudamore, Tertius A. Hough, Sara Wells, Frances M. Ashcroft, Michael A. McDonough, Christopher J. Schofield, Roger D. Cox

**Affiliations:** 1 MRC Harwell, Harwell Oxford Campus, Oxfordshire, Oxford, OX11 0RD, United Kingdom; 2 Chemistry Research Laboratory, University of Oxford, Mansfield Road, Oxford, OX1 3TA, United Kingdom; 3 Henry Wellcome Centre for Gene Function, Department of Physiology, Anatomy and Genetics, University of Oxford, Parks Road, Oxford, OX1 3PT, United Kingdom; Hospital Infantil Universitario Niño Jesús, SPAIN

## Abstract

In 2007, a genome wide association study identified a SNP in intron one of the gene encoding human FTO that was associated with increased body mass index. Homozygous risk allele carriers are on average three kg heavier than those homozygous for the protective allele. FTO is a DNA/RNA demethylase, however, how this function affects body weight, if at all, is unknown. Here we aimed to pharmacologically inhibit FTO to examine the effect of its demethylase function *in vitro* and *in vivo* as a first step in evaluating the therapeutic potential of FTO. We showed that IOX3, a known inhibitor of the HIF prolyl hydroxylases, decreased protein expression of FTO (in C2C12 cells) and reduced maximal respiration rate *in vitro*. However, FTO protein levels were not significantly altered by treatment of mice with IOX3 at 60 mg/kg every two days. This treatment did not affect body weight, or RER, but did significantly reduce bone mineral density and content and alter adipose tissue distribution. Future compounds designed to selectively inhibit FTO’s demethylase activity could be therapeutically useful for the treatment of obesity.

## Introduction

Single nucleotide polymorphisms (SNPs) within intron one of the *FTO* gene are associated with body mass index (BMI) in human populations[1]. The homozygous *FTO* risk allele (rs9939609, A allele) increases the risk of obesity by approximately 1.7 fold[1]. We, and others, have shown that knockout of *Fto* in mice leads to a lean phenotype[2–4] and that FTO overexpression leads to obesity[5]. Recently, it has been suggested that the element marked by the *FTO* intron 1 SNPs affect other genes nearby such as called *IRX3* or *RPGRIP1L*, rather than *FTO* itself[6,7]. However, these studies cannot rule out a role for the *FTO* gene, or the possibility that FTO expression is regulated by the obesity SNPs in particular cells and tissues or at particular developmental ages.

When *FTO* was first associated with an increased BMI its function was unknown. We predicted by sequence analysis that the FTO protein had a double-stranded beta-helix fold homologous to those of other Fe(II) and 2-oxoglutarate (2OG) dependent oxygenases, such as AlkB[8]. We also showed that FTO is capable of demethylating modified nucleic acids including *N* ^1^-methyladenine (m^1^A), *N* ^3^-methylcytosine (m^3^C) and has a preference for *N* ^3^-methylthymine (m^3^T) on single stranded nucleic acids[8]. Subsequently, FTO was shown to also act on *N* ^3^-methyluracil (m^3^U) and *N* ^6^-methyladenine (m^6^A) in RNA[9,10]. Evidence has been presented that FTO functions in processing pre-mRNA or other nuclear RNAs[10].

In animals, 2OG oxygenases have a wide range of functions including: DNA repair, post-translational modifications, the regulation of the hypoxic response, and fatty-acid metabolism[11]. 2OG oxygenases require non-haem ferrous iron as a cofactor, 2OG and molecular oxygen as co-substrates, and produce succinate and carbon dioxide as co-products in addition to a product resulting from two electron oxidation of the primary substrate. The *N*-terminal oxygenase domain of FTO contains the catalytic core which is vital for its oxygenase/demethylase activity; the role of the α-helical *C*-terminal domain of FTO is still not fully understood [12]. The oxygenase domain contains a highly conserved two histidine and one carboxylate iron-binding motif (residues His228/231, Asp230/233, His304/307; human/mouse sequences shown) and a pair of arginine residues (Arg313/316 and Arg319/322) which bind the Fe(II) co-factor and the 2OG co-substrate C-5 carboxylate, respectively[8,12]. Substitution of the Fe(II) binding residue His-304 to alanine reduces 2OG turnover and substitution of Arg-313 to alanine ablates activity[8].

**Fig 1:**
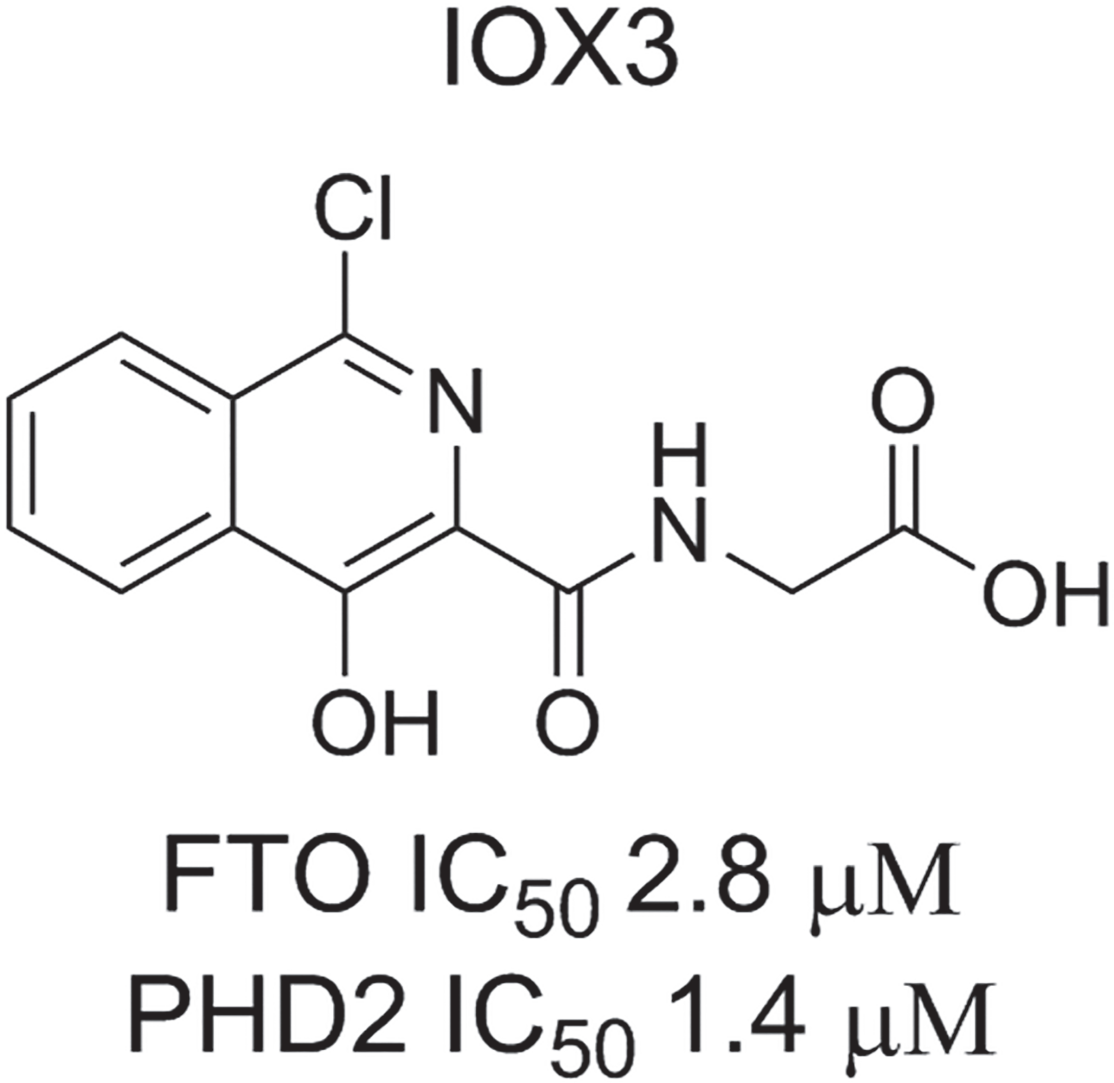
Chemical structure of IOX3 and IC_50_ values for FTO and PHD2.

There is an unmet clinical need for new approaches to treat obesity. In a screen for compounds that inhibit the demethylase activity of FTO, IOX3 was found to be a relatively potent inhibitor (IC_50_ 2.76 ± 0.9 μM) however it inhibits the hypoxia inducible factor prolyl-hydroxylases (PHDs) with similar potencies (1.4 μM for PHD2) (**Fig. 1**) [13,14]. IOX3 is a known inhibitor of the PHDs (isoforms PHD1-3) that are active in cells and animals[14,15]. Crystallographic analyses reveal that IOX3 binds at the active site of both FTO and the PHDs, occupying both the 2OG and the nucleotide binding sites[13]. Here we show for the first time that IOX3 treatment can effect fat localisation *in vivo*, and affect oxygen consumption *in* vitro.

## Materials and Methods

### Synthesis of IOX3

IOX3 [(1-chloro-4-hydroxy-isoquinoline-3-carbonyl)-amino]-acetic acid was prepared as described[16] and tested for purity [> 98%] by ^1^H-NMR, ^13^C-NMR and LC-MS.

### Pharmacological inhibition of FTO with IOX3 *in vitro*

C2C12 mouse myoblast cells (ATCC, Virginia, U.S.A) and mouse embryonic fibroblasts (MEFs) were treated for 16 hours with vehicle control [0.5% DMSO (Sigma-Aldrich, Dorset, U.K.) pH 7] or 1 μM IOX3 (dissolved in 0.5% DMSO pH 7).

### siRNA knockdown on *Fto in vitro*

Gene knockdown was achieved by treating C2C12 cells with siRNA (Invitrogen Stealth RNAi) and Lipofectamine 2000 (Invitrogen, California, U.S.A.).

### Seahorse extracellular flux analysis

Cells were plated onto Seahorse 24-well plates (Seahorse, Massachusetts, U.S.A.) and analysed within 24-hours. The cartridge was loaded with the compounds to test, [oligomycin, carbonyl cyanide-*4*-(trifluoromethoxy)phenylhydrazone (FCCP), and rotenone combined with antimycin A] and the XF24 instrument calibrated. Oxygen consumption rate (OCR) and extracellular acidification rate (ECAR) were measured continuously as previously described[17]. Briefly readings were collected every 9 min after a mixing, waiting and recording period in each well. Initially basal measurements were collected, before injection of oligomycin, then FCCP, and then with rotenone combined with antimycin A (AA). Values obtained were corrected for calcein staining intensity using a live/dead cell viability assay (Invitrogen, California, U.S.A.).

Liquid chromatography- mass spectrometry [LC-MS] for quantification of nucleosides

Samples were prepared and analysed as described in[18].

### Western blot analysis and antibodies

Western blots were performed on 10–60 μg of total proteins. A custom made recombinant rabbit anti mFTO antibody[3] was used to detect FTO and antibodies were purchased to detect phosphorylated AMPK alpha (#2535P cell signaling, Massachusetts U.S.A.) and HIF-1α (NB100-122 Novus Biologicals, Cambridge U.K.). The membranes were incubated with a secondary fluorescent (IRDye 800CW or IRDye 680RD) conjugated anti-source primary antibody (1:15,000; LI-COR, Nebraska, U.S.A.) for one hour at room temperature in complete darkness. Detection of the secondary output was performed using the Odyssey SA Infrared Imaging System (LI-COR Biosciences, Cambridge, UK).

### Ethics statement

Animal studies were carried out under the guidance issued by the MRC; Responsibility in the Use of Animals for Medical Research (July 1993) and Home Office Project Licence No. 30/2642. Mice were kept in accordance with U.K. Home Office welfare guidelines and project license restrictions under controlled light (12 h light and 12 h dark cycle), temperature (21 ± 2°C) and humidity (55 ± 10%) conditions. They had free access to water (25 ppm chlorine) and were fed *ad libitum* on a commercial diet (SDS maintenance chow, RM3, 3.6 kcal/g, Essex, U.K.).

### Mouse phenotyping

Forty C57BL/6J six-week old male mice housed in groups of five were weighed and then ranked and randomised by cage to evenly distribute mice of different weights to each dosing group. Mice were treated by oral gavage once every other day for 40 days with 10 mg/ml of IOX3 in 2% methylcellulose 5% DMSO pH 7 (60 mg/kg every 2 days) or an equivalent amount of vehicle (2% methylcellulose 5% DMSO pH 7).

Mice were weighed each week during the trial, and characterised using a standardised metabolic phenotyping pipeline. Phenotyping tests were performed according to EMPReSS (European Phenotyping Resource for Standardised Screens from EUMORPHIA) standardised protocols as described at http://empress.har.mrc.ac.uk. Body mass was measured on scales calibrated to 0.01 g. Analysis of body composition was performed by DEXA using the Lunar PIXImus Mouse Densitometer (Wipro GE Healthcare, Madison, U.S.A.) and/or with an Echo MRI whole body composition analyzer (Echo Medical System, Texas, U.S.A.).

Terminal blood samples were collected from mice aged 11.5 weeks as follows: Mice were fasted for 6 hours prior an intraperitoneal overdose of Euthatal. Blood samples were collected by retro-orbital puncture into paediatric lithium heparin tubes. Samples were kept on wet ice until being centrifuged for 10 min at 8000 x g in a refrigerated centrifuge set to 8°C. Plasma clinical chemistry was performed on a Beckman Coulter AU680 analyser using reagents and settings recommended by the manufacturer.

For pathological evaluation, a range of tissues were harvested from mice (https://www.mousephenotype.org/impress/protocol/101/7) fixed in 10% neutral buffered formalin and processed routinely to generate haematoxylin and eosin stained tissue sections. Tissue sections were evaluated microscopically and lesions scored semi-quantitatively [19].

### Statistical methods

Results are expressed as mean ± SEM. Comparisons between two groups were made by unpaired Student’s t-test and two-way analysis of variance (ANOVA) with repeated measures, as appropriate (GraphPad Prism, California, U.S.A.). P<0.05 was considered to be statistically significant.

## Results

### Pharmacological inhibition of FTO *in vitro*

It has been previously reported that IOX3 can significantly reduce oxygen consumption after 24 hours of treatment in cardiosphere-derived cells, with no impact on cell proliferation[20]. To further investigate the effect of IOX3, we used the Seahorse XF24 apparatus to measure oxygen consumption rate (OCR, a measurement of oxidative metabolism) and extracellular acidification rate (ECAR, a measurement of glycolysis) in vehicle and 1 μM IOX3-treated C2C12 muscle myoblast cells.

**Fig 2:**
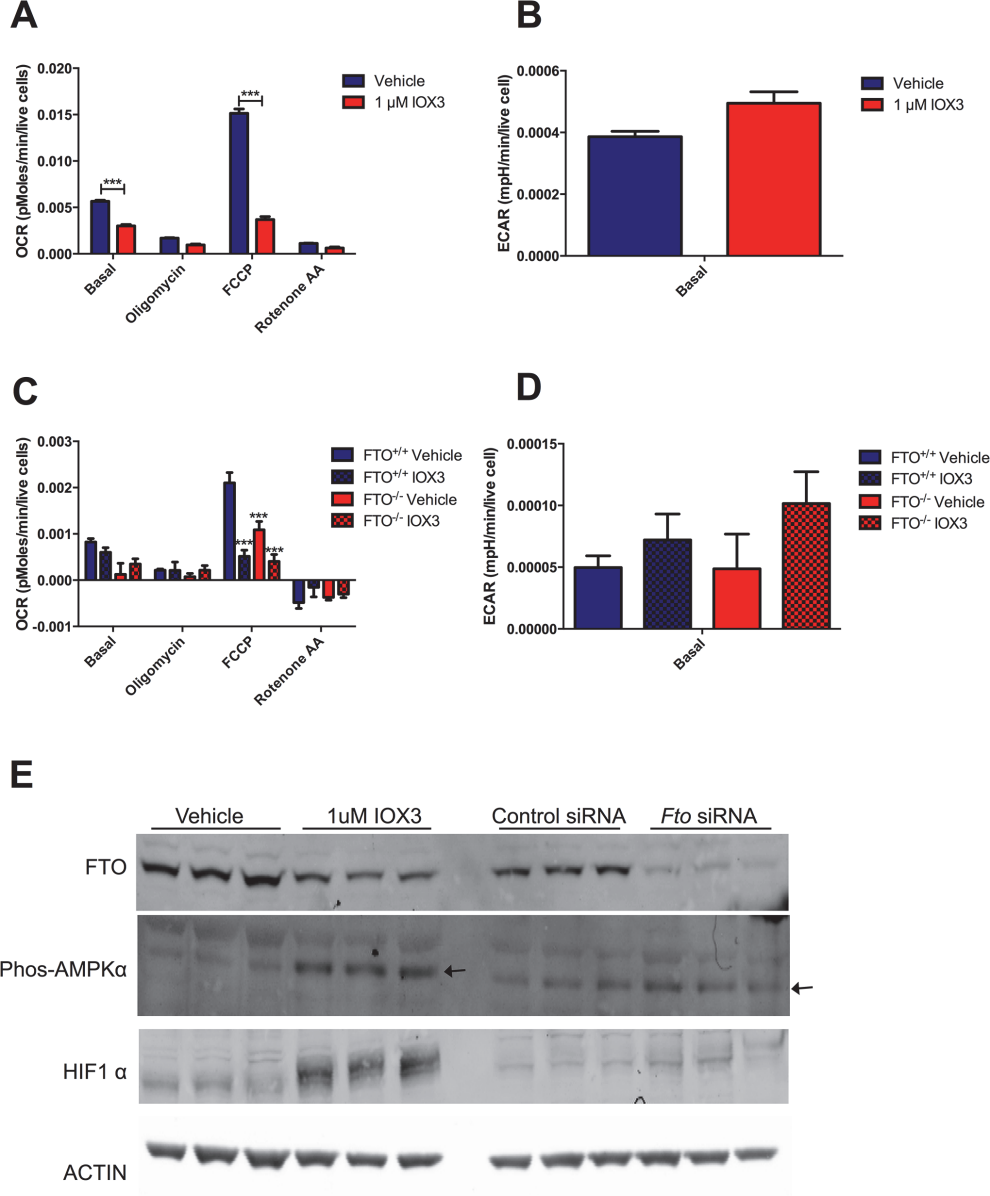
Oxygen Consumption Rate (OCR), Extracellular Acidification Rate (ECAR) of C2C12, and wild-type and FTO knockout MEFs treated with 1 μM IOX3 or an equivalent amount of vehicle control for 16 hours. A) OCR and, B) basal ECAR measured in C2C12 cells treated with vehicle (n = 10) and 1 μM IOX3 (n = 10) at baseline and after Oligomycin, FCCP and Rotenone treatment, data normalised to live stain. C) OCR and, D] ECAR measured in *Fto* ^*+/+*^ and *Fto* ^*-/-*^ MEFs cells treated with vehicle and 1 μM IOX3 (n = 5 per group) at baseline and after Oligomycin, FCCP and Rotenone treatment, data normalised to live stain. Data were analysed using a 2 way ANOVA with Bonferroni post-hoc test. Data is of readings following each compound injection and are expressed as mean ± SEM. E) Expression of FTO, phosphorylated-AMPKα and HIF-1α with representative ACTIN in cells treated with vehicle, 1uM IOX3, control scrambled siRNA or *Fto* siRNA for 24 hours. N = 3 biological replicates per condition.

Data collected for OCR and ECAR (**Fig. 2 A,B**) were normalised to the intensity of live cell staining. Basal OCR was significantly reduced in IOX3-treated C2C12 cells (**Fig. 2A**), and there was a trend for increased basal ECAR (**Fig. 2B**), which together suggests an increase in glycolytic metabolism. Addition of oligomycin reduced OCR from baseline in both vehicle and IOX3-treated cells (**Fig. 2A**). The mitochondrial uncoupler FCCP, which is used to identify the maximal respiration rate, had significantly less effect on OCR levels in IOX3-treated cells (3.8-fold increase) than the vehicle group (8.9-fold increase). The results indicate that IOX3-treated cells have a reduced maximal respiration and spare respiratory capacity. After addition of rotenone and antimycin A, which inhibit complex I and complex III of the electron transport chain respectively, OCR levels dropped in both groups and there was no significant difference in the remaining non-mitochondrial respiration levels.

As IOX3 does not specifically inhibit FTO, and has been shown to act on the PHDs and other 2OG oxygenases[14], we assessed the proportion of the differences in **Fig. 2A** that were due to inhibition of FTO using mouse embryo fibroblasts (MEFs) from wild-type and *Fto* ^-/-^ mice[21] treated with vehicle or IOX3 for 16 hours. There was no significant difference in basal OCR between vehicle and IOX3-treated wild-type or *Fto* ^*-/-*^ MEFs [**Fig. 2C**]. There was also no significant difference in OCR after oligomycin treatment. Treatment with FCCP revealed a reduced maximal respiratory capacity in *Fto* ^*-/-*^ vehicle-treated MEFs compared to wild-type vehicle-treated MEFs (52% of the wild-type maximal capacity). Treatment of either genotype with 1 μM IOX3 significantly reduced the maximal respiratory capacity (FCCP treatment) of the MEFs, compared to vehicle-treated wild-type MEFs. The IOX3 treatment of wild-type MEFs reduced maximal respiratory capacity by 76% (**Fig. 2C**), which is 24% more than knockout of FTO alone (see above). The difference between wild-type and knockout cells treated with IOX3 was not significantly different. Overall the reduction of maximal respiratory capacity in *Fto* ^*-/-*^ MEFs suggests that some of the reduction in C2C12 cells is likely due to the IOX3 inhibiting FTO and the rest is due to off-target effects of IOX3 (likely involving activation of the HIF system via PHD inhibition) (**Fig. 2C**). ECAR levels were not significantly different between any of the groups, although there was a trend for increased basal ECAR in IOX3-treated wild-type and *Fto* ^-/-^ MEFs (**Fig. 2D**).

C2C12 cells treated with 1 μM IOX3 for 24 hours showed a 51% decrease in FTO protein expression (P = 0.00102) compared to vehicle-treated cells (**Fig. 2E**). Cells treated with *Fto* siRNA showed an 81% reduction in FTO protein expression (**Fig. 2E**). In order to assess the effect of IOX3 on hypoxia signalling pathways, cells were treated with a single dose of 1 μM IOX3. This resulted in an increase in phosphorylated AMPKα (3.45 fold, P = 0.00116) and HIF-1α (4.21 fold, P = 0.00585). Knockdown of FTO by siRNA did not significantly alter the levels of AMPKα or HIF-1α (**Fig. 2E**). As FTO has been shown to catalyse demethylation of m^6^A bases, we investigated the effect of IOX3 on the ratio of m^6^A to adenosine in C2C12 cells treated with the 1 μM IOX3 or vehicle for 24 hours: no significant difference was observed under the tested conditions (**S1 Fig.**, P = 0.13).

### Pharmacological inhibition of FTO *in vivo*

**Fig 3:**
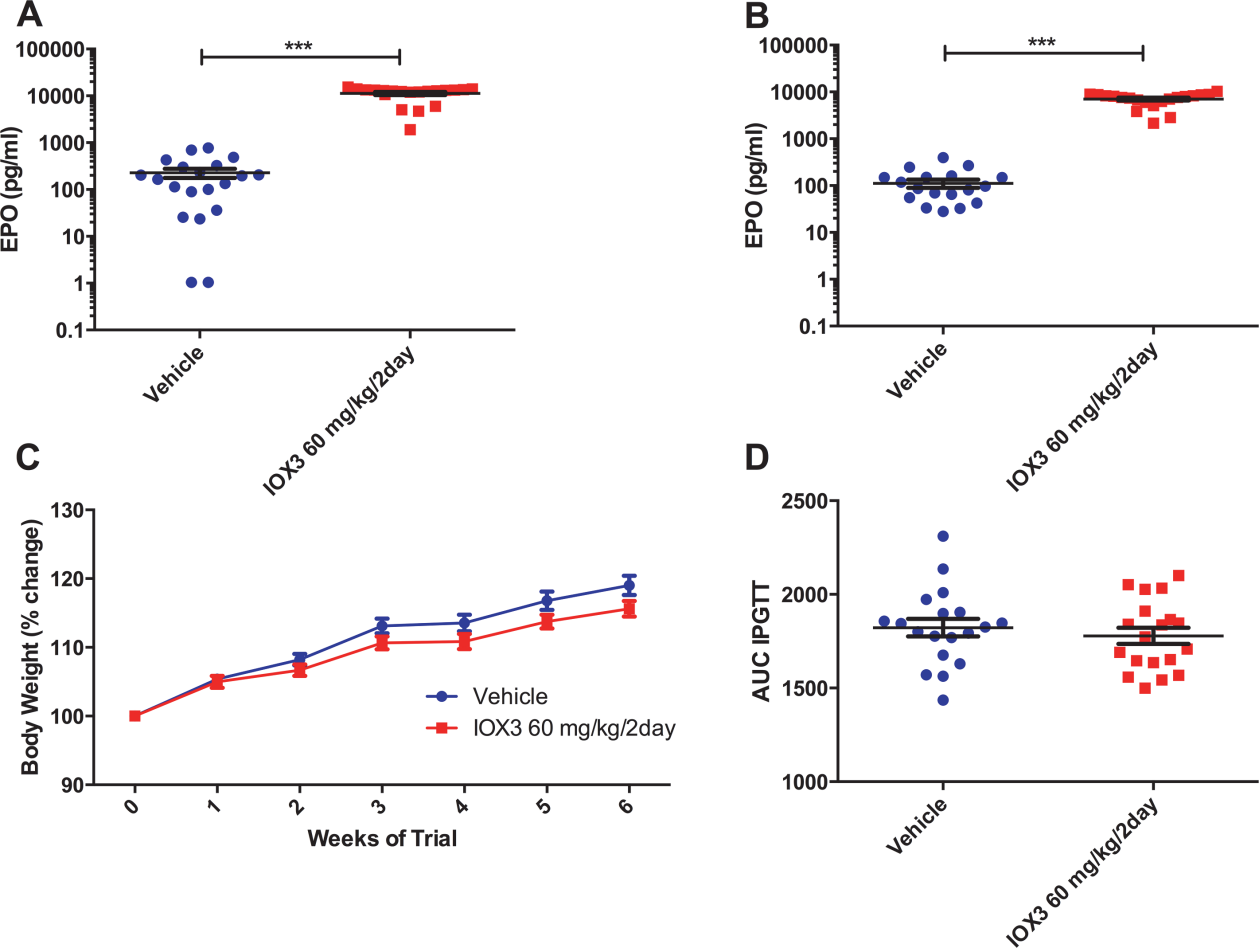
Plasma EPO levels in vehicle and 60 mg/kg/2days IOX3 treated mice and the effect on FTO protein levels. A) Plasma EPO levels after one week of dosing in vehicle (n = 20) and 60 mg/kg every two days IOX3 (n = 20) treated mice. B) Terminal plasma EPO levels at 40 days after beginning of the trial, vehicle (n = 20) and 60 mg/kg every two days IOX3 (n = 20) treated mice. C) Weekly body weight of Vehicle (n = 20) and 60 mg/kg every two days IOX3 (n = 20). D) Area under the curve calculated from blood glucose curves. Data were analysed using a student’s t-test and time course data were analysed by 2-way ANOVA with Bonferroni post-hoc test *P<0.05, **P<0.01, ***P<0.001. Data are expressed as mean ± SEM.

As anticipated, mice treated with IOX3 (60 mg/kg every two days) had significantly increased levels of erythropoietin (EPO) one week after dosing (P = 7.2E-16 **Fig. 3A**) and EPO remained at a high level during the 40 days of dosing (P = 4.70E-17, **Fig. 3B**) compared with vehicle-treated mice. This observation is consistent with previous reports that IOX3 acts as a PHD inhibitor in mice and can cause erythropoiesis[22,23]. Levels of FTO protein in whole liver, brain and gastrocnemius muscle were unaffected in tissue collected at the end of the trial (**S2 Fig.**).

There was no significant difference in body-weight between vehicle and IOX3-treated (60 mg/kg every two days) animals during the trial (**Fig. 3C**). There was no significant difference in fat mass or lean mass during the trial (**S3A,B Fig.**). Food intake over a 24-hour period was not significantly different between the groups (**S3C Fig.**). Indirect calorimetry revealed no significant differences in oxygen consumption, carbon dioxide production, respiratory exchange ratio (RER) or energy expenditure in these animals (**S3D-G Fig.**). An intraperitoneal glucose tolerance test revealed no significant differences between the groups (**Fig. 3D**).

**Fig 4:**
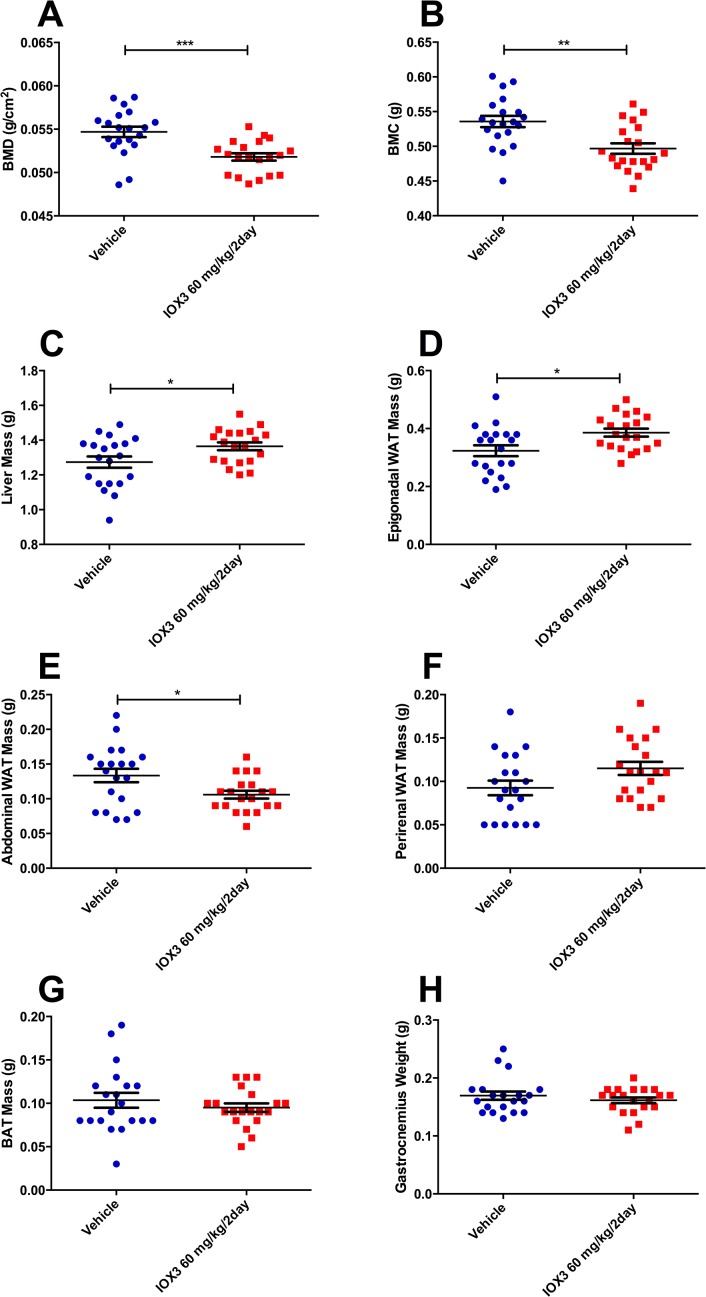
DEXA and organ weights of vehicle and 60 mg/kg every two days IOX3-treated mice. A) Bone Mineral Density (BMD), B) Bone Mineral Content (BMC), C) Liver mass, D) Epigonadal white adipose tissue (WAT), E) Abdominal WAT, F) Peri-renal WAT, G) Brown adipose tissue (BAT), H) Calf muscle weight of vehicle (n = 20) and 60 mg/kg every two days IOX3 (n = 20) treated mice. Data analysed by student’s t-test *P<0.05, **P<0.01, ***P<0.001. Data are expressed as mean ± SEM and individual data points are shown.

Bone mineral density (BMD) and bone mineral content (BMC) were both significantly lower in the IOX3-treated (60 mg/kg every two days) mice (P = 3.46E-4 and P = 1.15E-3 respectively **Fig. 4A,B**), but absolute liver mass was significantly increased (7% increase, P = 0.0276, **Fig. 4C**). The liver weight as a percentage of body mass was also significantly different, with 4.68 ± 0.12% for vehicle-treated mice and 5.01 ± 0.06% for animals treated with IOX3 (60 mg/kg every two days, P = 0.0232).

IOX3-treated (60 mg/kg every two days) mice had significantly increased epigonadal fat weight compared to vehicle-dosed controls (P = 0.0105), but significantly lower abdominal white adipose tissue (WAT) weight (P = 0.0186, **Fig. 4D,E**), which indicates that IOX3-treated mice have altered fat distribution. Peri-renal WAT, brown adipose tissue (BAT) and gastrocnemius weights were not significantly different between treatment groups (**Fig. 4F,G,H**).

In terminal plasma there was no significant difference in circulating leptin, leptin levels adjusted for fat mass, or plasma insulin (**S4A–C Fig.**) between IOX3-treated (60 mg/kg every two days) and vehicle-treated mice. However, terminal plasma of IOX3-treated mice had significantly higher plasma urea (P = 2.73E-8), creatinine (P = 1.77E-6), ALP (P = 4.36E-7), free fatty acids (p = 0.0202) and iron (P = 9.83E-5), and significantly decreased levels of chloride (P = 1.77E-6), calcium (P = 0.0372), creatinine kinase (P = 0.0371) and uric acid (P = 4.75E-4) (**Table 1**).

**Table 1:** Plasma Biochemistry vehicle (n = 20) and 60 mg/kg/2days IOX3 (n = 20) treated mice.

**Assay**	**Units**	**Vehicle**		**IOX3 60 mg/kg/2days**		**P-value**
		**Mean**	**SEM**	**Mean**	**SEM**	
Sodium	mmol/l	143.950	0.407	143.167	0.326	NS
Potassium	mmol/l	6.488	0.456	5.738	0.245	NS
Chloride	mmol/l	106.050	0.285	103.556	0.336	***
Urea	mmol/l	8.522	0.162	10.716	0.274	***
Creatinine	μmol/l	11.960	0.381	17.760	1.040	***
Calcium	mmol/l	1.816	0.056	1.660	0.045	*
Inorganic Phosphate	mmol/l	2.752	0.157	2.280	0.071	NS
ALP	U/l	79.675	2.083	99.055	2.410	***
ALT	U/l	30.035	5.485	33.930	5.448	NS
AST	U/l	70.155	6.646	75.030	9.250	NS
Total Protein	g/l	45.440	0.456	42.885	1.418	NS
Albumin	g/l	23.370	0.317	22.640	0.390	NS
Total Cholesterol	mmol/l	2.150	0.076	2.348	0.068	NS
HDL	mmol/l	1.310	0.048	1.420	0.045	NS
LDL	mmol/l	0.421	0.017	0.456	0.012	NS
Glucose	mmol/l	17.516	1.521	15.551	1.005	NS
Triglycerides	mmol/l	0.760	0.096	0.682	0.063	NS
Glycerol	μmol/l	319.525	46.491	200.440	11.845	NS
Free Fatty Acids	mmol/l	0.666	0.043	0.689	0.020	*
Total Billirubin	μmol/l	4.115	0.360	4.038	0.284	NS
LDH	U/l	717.905	69.157	555.617	74.673	NS
Iron	μmol/l	21.450	0.783	28.165	1.330	***
Amylase	U/l	577.435	17.058	563.675	28.378	NS
CK	U/l	159.760	35.694	48.240	2.625	*
Uric Acid	μmol/l	186.260	26.578	92.889	4.401	***
Fructosamine	μmol/l	189.895	2.931	186.625	5.897	NS

Abbreviations: Alkaline Phosphatase (ALP), Alanine Transaminase (ALT), Aspartate Aminotransferase (AST), High-Density Lipoprotein (HDL), Low-Density Lipoprotein (LDL), Lactate Dehydrogenase (LDH), Creatine Kinase (CK). One way ANOVA Bonferroni-corrected for multiple comparisons. *P<0.05, **P<0.01, ***P<0.001. SEM, Standard Error of the Mean; NS, Not Significant.

No significant pathology was observed on microscopic evaluation of organs (including liver and kidneys) from mice treated with 60 mg/kg IOX3 every two days.

## Discussion

Treatment of mice with IOX3 at 60 mg/kg every two days did not affect body weight or RER, but it did alter fat pad weights and bone mineral density and content. However, because IOX3 also inhibits other 2OG oxygenases, in particular the HIF PHDs [14], it is quite likely that HIF-related effects are responsible, at least in part, for the observed phenotypic effects.

### FTO inhibition

Global adult onset knockout of *Fto* removes the high lethality of germline loss, and causes a reduction in weight, primarily as loss of lean mass at 9 weeks of age, and a later convergence of weight driven by an increase in fat mass[21]. These mice have a reduction in RER with no significant change in food intake or energy expenditure[21]. Partial inhibition of FTO’s demethylase activity by treating mice with IOX3 60 mg/kg every 2 days does not fully copy the phenotype observed for the adult onset knockout of *Fto*. This could be due to a number of factors, and phenotypic effects may require an increased dose. Protein levels of FTO were not significantly altered *in vivo* by the current dose. We do not know the half-life of the drug in the mouse, so the dosing regimen may not have kept levels in the plasma sufficiently high enough to cause a significant inhibitory effect on FTO. Hsieh and colleagues dosed mice with 60 mg/kg intravenously into the tail vein and saw an increase in EPO levels of 100-fold four hours after treatment with IOX3 [22], which is similar to the fold increase we saw after 1 week and after 40 days (**Fig. 3A,B**). This suggests that there is not an increased effect with intravenous administration. Hsiesh and colleagues also dosed rhesus macaques 60 mg/kg twice weekly over a period of 6–8 weeks and saw no side-effects, which indicated to us that it may be sufficient to dose mice on alternate days.

An FTO R316Q substitution is responsible for an autosomal-recessive lethal syndrome in a large Palestinian Arab consanguineous multiplex family[24]. Skin fibroblasts cultured from one FTO^R316Q^ patient showed an altered cell morphology, decreased proliferation and increased expression of the senescence-associated β-galactosidase[24]. This is similar to observations by Gulati and colleagues that saw decreased proliferation in *Fto* ^*-/-*^ MEFs compared to wild-type controls[25]. In the IOX3-treated C2C12 cells we also see decreased levels of live cells after 16 hours of treatment (**S5A Fig.**). The *Fto* ^*-/-*^ MEFs treated with IOX3 showed significantly less live staining than wild-type vehicle and IOX3 treated MEFs, but there was no significant difference in vehicle and IOX3-treated wild-type MEFs (**S5B Fig.**). This suggests that the *Fto* ^*-/-*^ MEFs and the C2C12s are more sensitive to IOX3 treatment than wild-type MEFs or other cell-types previously examined[20]. Unexpectedly, IOX3 treatment of C2C12 cells results in a significant reduction in FTO protein that was not observed *in vivo* in IOX3-treated mice. This difference may reflect the *in vivo* pharmacokinetic properties of IOX3.

When C2C12 cells are treated with IOX3 we do not observe a significant effect on global levels of m6A in the cell (**S1 Fig.**). Although this could indicate that IOX3 is not altering FTO function, previous *in vivo* experiments have also reported that altering expression of FTO does not alter cellular m6A composition[18,26].

### Effects of prolyl-hydroxylases inhibitors

Inhibition of the HIF prolyl-hydroxylases stabilises HIF, leading to activation of the HIF pathway, which is normally stimulated during hypoxic conditions[27,28]. It has also been shown that stabilisation of HIF-1α mediates adaptation to hypoxia by downregulating mitochondrial oxygen consumption, thereby shunting glucose to glycolysis to maintain ATP production[29,30]. Previous studies have shown that *in vitro* use of other prolyl-hydroxylases inhibitors (PHIs) such as dimethyloxalylglycine (DMOG), a promiscuous 2OG oxygenase inhibitor[15,31], also decreases oxygen consumption rate and increase extracellular acidification rate, suggesting that these compounds may downregulate mitochondrial oxidative phosphorylation and increase levels of glycolysis[32]. IOX3 has also previously been shown to reduce oxygen consumption in cells[20], an effect that we were able to replicate in C2C12 myoblast cells. We also treated wild-type and *Fto* ^*-/-*^ MEFs to see how much of this was due to inhibition of FTO. When treated with the uncoupler FCCP, to determine maximal respiratory capacity, both wild-type and *Fto* ^*-/-*^ MEFs treated with IOX3 had a significantly lower OCR than wild-type vehicle-treated cells. Vehicle-treated *Fto* ^*-/-*^ MEFs had an intermediate phenotype, with a higher OCR than the IOX3-treated cells but one lower than vehicle-treated wild-type MEFs. This suggests that a proportion (approximately 52%) of the maximal respiratory capacity of the cells is affected by FTO inhibition or deletion in MEFs.

The PHDs that regulate degradation of HIF-α are not the only prolyl-hydroxylases[15]. Other PHDs include OGFOD1 (a ribosomal protein prolyl-3-hydroxylase[33–35]), and the collagen prolyl-4-hydroxylases[36], some of these are HIF regulated[37]. The collagen prolyl-hydroxylases have a strong requirement for vitamin C for activity, and vitamin C deficiency is believed to cause scurvy *via* impaired collagen prolyl-4-hydroxylase activity. Prolyl-3-hydroxylases also catalyse collagen hydroxylation, deletion of Prolyl-3-hydroxylase1 causes osteogenesis imperfecta, or brittle bone disease[38]. Our IOX3-treated mice had lower BMD and BMC than vehicle-treated controls. Administration of EPO has previously been shown to increase bone formation *in vivo* via the Jak-Stat signalling pathway[39]. However, this was studying mice dosed with EPO rather than a PHI and other experiments suggest that PHIs can act to reduce osteoclast differentiation[40]. Osteoclasts are important for bone resorption, and we do observe significantly lower calcium and inorganic phosphate in the plasma of the 60 mg/kg/2days IOX3-treated mice, which could suggest a decrease in bone resorption. BMD and BMC are significantly lower in the global *Fto* ^*-/-*^ mice (unpublished in-house data) but we did not observe a BMD or BMC effect when *Fto* was knocked out in the adult mouse[21], suggesting this phenotype has an embryonic or early postnatal origin. In addition to the PHDs, the other prolyl-hydroxylases[14,15] also have important roles, including in collagen and bone formation. Thus, if IOX3 can also inhibit, for example, collagen prolyl-3-hydroxylase 1, it could cause alterations in BMC and BMD as observed in our mice[38].

### IOX3 effects

As IOX3 inhibits the HIF PHDs[14,22,41,42], and so affects HIF targets, we anticipated off-FTO-target effects *in vivo*, such as the observed increases in plasma EPO. Plasma clinical chemistry results from mice treated with 60 mg/kg IOX3 every two days for the duration of the trial also demonstrate an unexpected adverse effect on renal function (increased urea and creatinine and decreased uric acid and chloride, see **Table 1**), although there was no evidence of morphological renal pathology in mice treated with either dose of IOX3 (data not shown).

FTO is highly expressed in the brain; Gao and colleagues used a *Nestin-*Cre line to induce *Fto* knockout in the nervous system, replicating the phenotype of the whole body *Fto* knockout[4]. Recently it has also been shown that *Fto* ^*-/-*^ mice have a diminished response to cocaine dosing, suggesting a role for FTO in D2R and mediated signalling[43]. Dopaminergic signalling is important to locomotor function, reward, and cognition, so an altered reward response could lead to increased/decreased weight gain. We do not know if IOX3 can penetrate the blood brain barrier (BBB). However in one study IOX3 is given to mice via tail vein injection (20 mg/kg), and is able to upregulate HIF in brain tissue[23]. This suggest that at our dose, 60 mg/kg every two days, a small proportion may get past the BBB, but whether this would be enough to cause any of the phenotypes we observe is unknown.

## Conclusion

Our study demonstrates that dosing cells with IOX3 can reduce FTO protein expression and reduce maximal respiration rate, a significant proportion of which is due to inhibiting FTO and the rest to other targets, *in vitro*. Although we cannot know which phenotypic effects of IOX3 treatment are due to inhibition of FTO and which are due to inhibition of other 2OG oxygenases including the PHDs, future compounds designed to selectively inhibit FTOs demethylase activity could be therapeutically useful for the treatment of obesity.

## Supporting Information

S1 Fig Levels of m^6^A to A in C2C12 cells treated with either vehicle or 1 μM IOX3 for 16 hours. (TIF)

S2 Fig FTO Protein expression in whole liver, brain and gastrocnemius muscle in vehicle and 60 mg/kg every two days IOX3 treated mice after the 40 day dosing trial. (TIF)

S3 Fig Additional data for IOX3 60 mg/kg/2days dosed mice. A) Weekly Fat mass and, B) Lean mass percentage change from baseline during the trial of vehicle (n = 20) and IOX3 60 mg/kg/2days treated mice (n = 20). Data were analysed using a 2 way ANOVA with bonferroni post-hoc test. C) Food intake over a 24 hour period, data analysed by student’s t-test. D) Volume of Oxygen (VO_2_) consumed, E) Volume of carbon dioxide (VCO_2_) produced, F) Respiratory exchange Ratio (RER), G) Energy Expenditure. Indirect calorimetry data was analysed with lean weight correction. Data are expressed as mean ± SEM.
(TIF)

S4 Fig Terminal plasma analysis of IOX3 60 mg/kg/2day dosed mice A) Leptin, B) Leptin corrected for fat mass and, C) Insulin. Data analysed by student’s t-test, and are expressed as mean ± SEM. (TIF)

S5 Fig Viability of cells treated with 1 μM IOX3 or an equivalent amount of vehicle control for 16 hours. Live stain (Calcein) of A) C2C12 cells and, B) wild-type and FTO knockout MEFs on cells after Seahorse XF24 measurements (**Fig. 2A,C**). Data analysed by student’s t-test *P<0.05, **P<0.01, ***P<0.001. Data are expressed as mean ± SEM.
(TIF)
