# Supplementary figures and images for: Pharmacological Inhibition of FTO
Source: PLoS One. 2015 Apr 1;10(4):e0121829. doi: 10.1371/journal.pone.0121829 (PMC4382163; doi:10.1371/journal.pone.0121829)

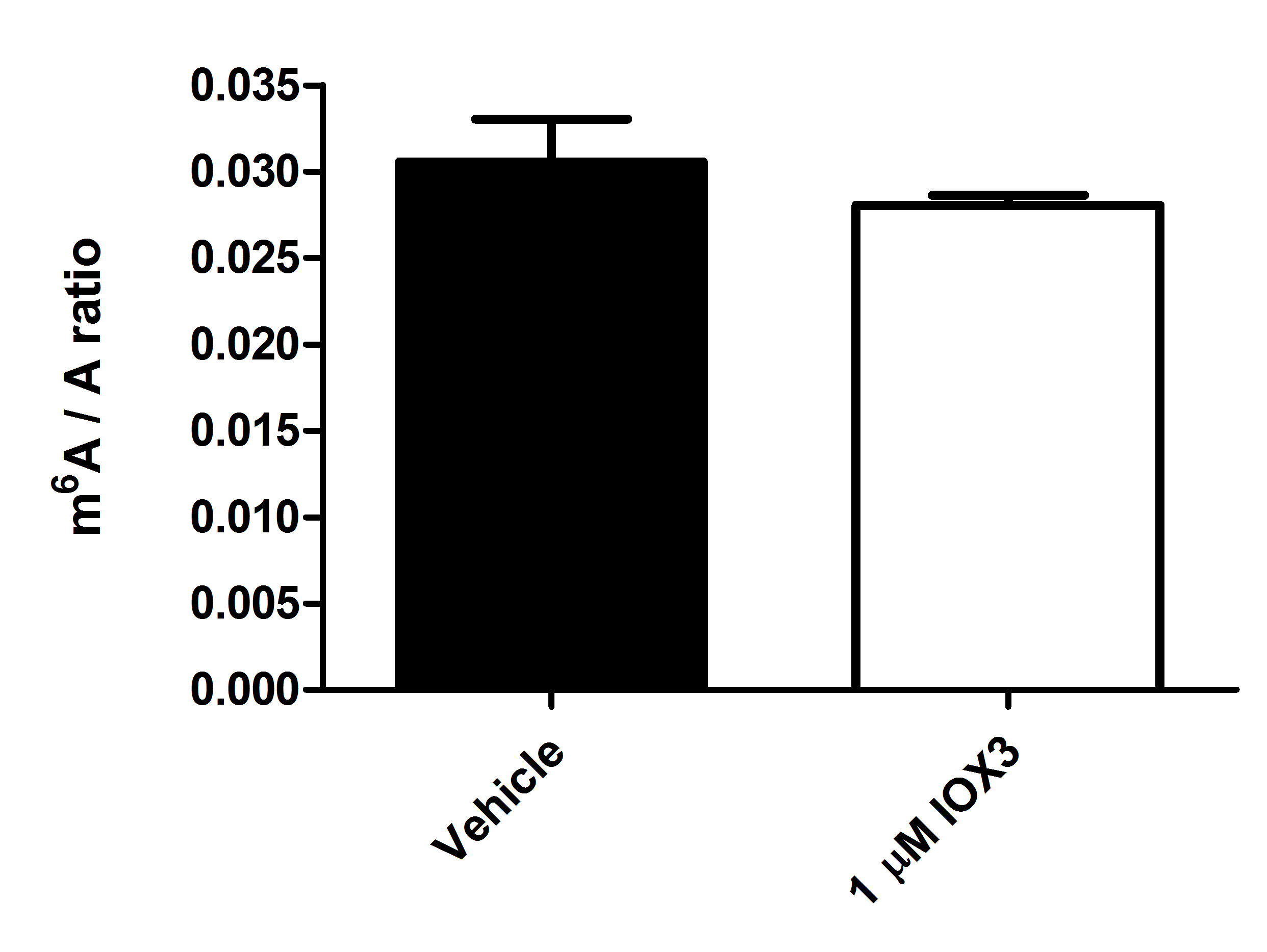

Supplement: S1 Fig — (TIF) [file pone.0121829.s001.tif]

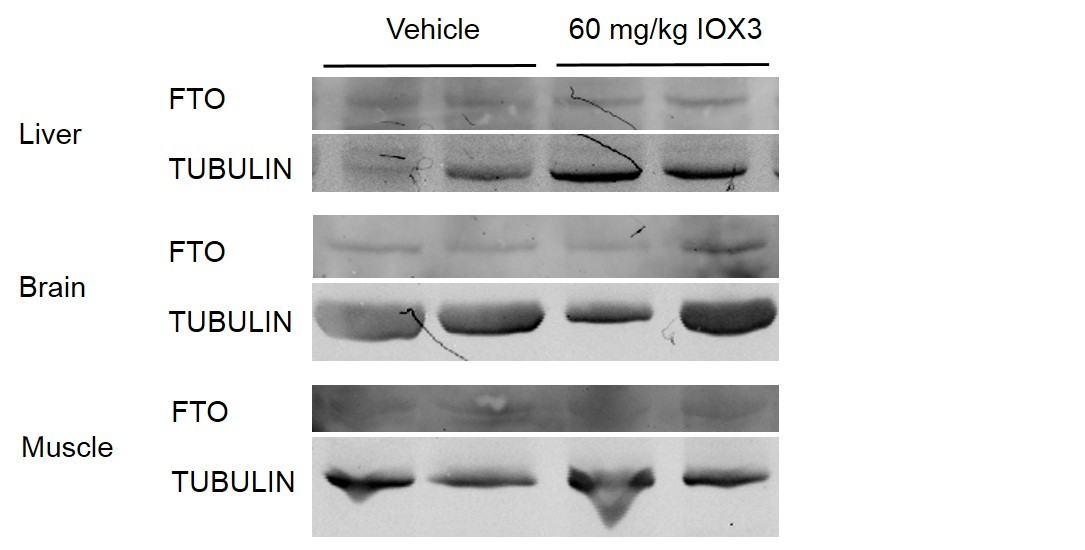

Supplement: S2 Fig — (TIF) [file pone.0121829.s002.tif]

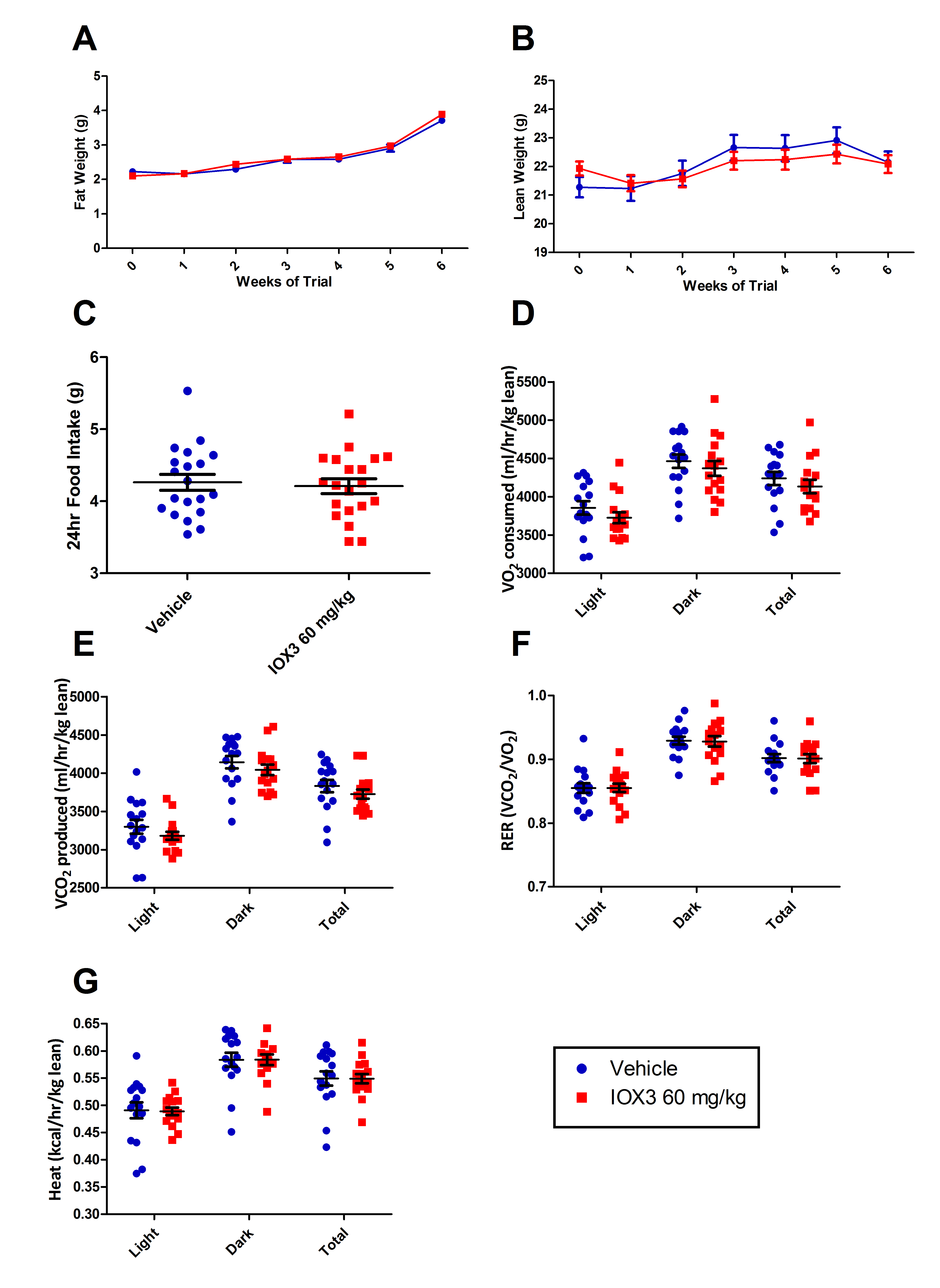

Supplement: S3 Fig — A) Weekly Fat mass and, B) Lean mass percentage change from baseline during the trial of vehicle (n = 20) and IOX3 60 mg/kg/2days treated mice (n = 20). Data were analysed using a 2 way ANOVA with bonferroni post-hoc test. C) Food intake over a 24 hour period, data analysed by student’s t-test. D) Volume of Oxygen (VO2) consumed, E) Volume of carbon dioxide (VCO2) produced, F) Respiratory exchange Ratio (RER), G) Energy Expenditure. Indirect calorimetry data was analysed with lean weight correction. Data are expressed as mean ± SEM. (TIF) [file pone.0121829.s003.tif]

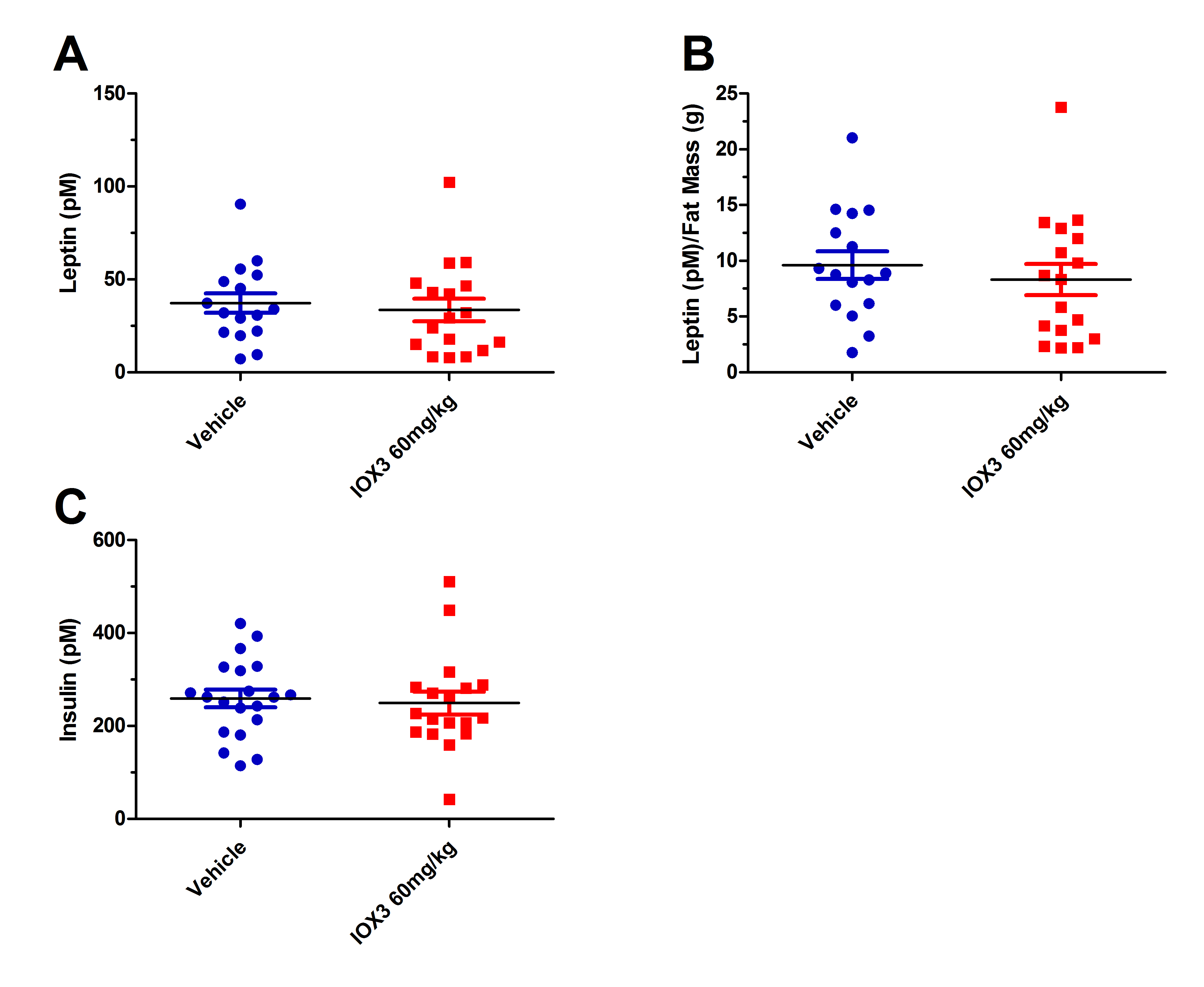

Supplement: S4 Fig — (TIF) [file pone.0121829.s004.tif]
